# Complete Genome Sequences of *Mandrillus leucophaeus* and *Papio ursinus* Cytomegaloviruses

**DOI:** 10.1128/genomeA.00781-15

**Published:** 2015-08-06

**Authors:** Earl Linwood Blewett, Carly J. Sherrod, Jordan R. Texier, Tom M. Conrad, Dirk P. Dittmer

**Affiliations:** a Department of Biochemistry and Microbiology, Center for Health Sciences, Oklahoma State University, Tulsa, Oklahoma, USA; b Department of Microbiology and Immunology, University of North Carolina School of Medicine, Lineberger Comprehensive Cancer Center, Chapel Hill, North Carolina, USA; c University of North Carolina, Curriculum in Biostatistics, Chapel Hill, North Carolina, USA

## Abstract

The complete genome sequences of *Mandrillus leucophaeus* and *Papio ursinus* cytomegaloviruses were determined. An isolate from a drill monkey, OCOM6-2, and an isolate from a chacma baboon, OCOM4-52, were subjected to pyrosequencing and assembled. Comparative alignment of published primate cytomegaloviruses (CMVs) showed variable sequence conservation between species.

## GENOME ANNOUNCEMENT

Cytomegaloviruses (CMVs) are members of the *Betaherpesvirinae* subfamily and infect most primates, including humans, Old World monkeys (OWM), and New World monkeys (NWM) (1). CMVs are considered to be host restrictive due to the lack of evidence of cross-species infection and are thought to have co-evolved with their host. Nonhuman-primate CMVs are the closest relatives to human CMV (HCMV) (2). Drill monkeys and baboons are infected in the wild and are also used as models for CMV vaccine and antiviral development (2). To solidify the evolutionary relationship between primate CMVs and HCMVs, a drill monkey CMV (DrCMV) isolate, OCOM6-2 (3), and a chacma baboon CMV (BaCMV) isolate, OCOM4-52 (4), were sequenced using a Roche GS Junior (454 Life Sciences, a Roche company, Branford, CT). The long read lengths (median, 380 nucleotides [nt]) allowed the complete CMV genomes to be *de novo* assembled using the 454 Assembly Suite, version 2.9, without manual intervention. CLC Genomics Workbench version 7.0.4 (CLC Bio-Qiagen, Aarhus, Denmark) was used for validation. A total of 82,066 reads were used for the DrCMV isolate with a Q40PlusBases score of 99.01%. A total of 111,885 reads were used for the BaCMV isolate with a Q40PlusBases percentage of 98.78% of all nucleotides. Annotations were transferred from the cercopithecine herpesvirus 5 strain 2715 (CHV5) (NCBI reference sequence NC_012783), using the Rapid Annotations Transfer Tool (RATT) (5) and verified using CLC Genomics Workbench version 7.0.4. CHV5 is a species-specific CMV most closely related to the sequenced isolates. Using the multiple sequence alignment program MAFFT shows that DrCMV and BaCMV share 78%, human CMV and chimpanzee CMV share 60%, and BaCMV and rhesus monkey CMV share 58% sequence identity across their entire genomes (6).

### Nucleotide sequence accession numbers.

The annotated sequences for *Mandrillus leucophaeus* isolate OCOM6-2 and *Papio ursinus* isolate OCOM4-52 cytomegaloviruses have been deposited in GenBank under the accession numbers KR297253 and KR351281, respectively.
